# Understanding the influence of sward type and dairy cow breed on enteric methane emissions through investigation of the rumen microbiome

**DOI:** 10.3389/fmicb.2026.1799911

**Published:** 2026-06-17

**Authors:** C. Dwan, F. Buckley, A. Das, P. W. O’Toole, T. F. O’Callaghan, D. Meehan, B. Horan, H. Costigan, A. Jezequel, B. Lahart

**Affiliations:** 1 Teagasc, Animal and Grassland Research and Innovation Centre, Fermoy, Ireland; 2 School of Biological, Earth and Environmental Sciences, University College Cork, Cork, Ireland; 3 School of Microbiology and APC Microbiome Ireland, University College Cork, Cork, Ireland; 4 School of Food and Nutritional Sciences, University College Cork, Cork, Ireland; 5 School of Agriculture and Food Science, University College Dublin, Dublin, Ireland

**Keywords:** clover, Holstein-Friesian × Jersey crossbred, methanogen, multispecies sward, pasture, rumen fermentation characteristics

## Abstract

The current study investigated the rumen microbiome of Holstein-Friesian (HF) and Holstein-Friesian × Jersey crossbred (JFX) dairy cows grazing three sward systems; a perennial ryegrass (*Lolium perenne*) monoculture receiving 250 kg nitrogen (N)/ha/year (PRG), perennial ryegrass with white clover receiving 125 kg N/ha/year (PRGWC), and a multispecies sward, consisting of grasses, clovers and herbs which also received 125 kg N/ha/year (MSS). Rumen fluid samples were collected at two time points, early-August and mid-October. Sward system had no effect on microbiome alpha or beta diversity. The bacterial genera *Lachnospira* and *Prevotellaceae Ga6A1 group* were both more abundant in PRGWC and MSS compared with PRG while *Pseudoramibacter* was more abundant in MSS compared with the other two sward systems. There was no difference in the total abundance of methanogenic archaea between swards (expressed as the ratio of archaea to bacteria) although the *Methanosphaera* genus was more abundant and the *Methanobrevibacter* genus was less abundant in PRGWC and MSS compared with PRG. The analysis also revealed a minor difference in microbiome beta diversity between the two dairy cow breeds, reflecting global microbiome configuration differences. Four specific bacterial genera were less abundant in JFX compared with HF. The JFX cows also had slightly greater *Methanobrevibacter* and slightly lower *Methanosphaera* abundance compared with the HF cows although total methanogen abundance was not different. The results from this study demonstrate that increasing sward species diversity has limited influence on the core rumen microbiome while crossbreeding HF with Jersey did have some influence. Both factors also altered the composition of the rumen methanogenic community. Further research is required to understand the relationship between these alterations and enteric methane emissions.

## 1 Introduction

The ability of ruminants to produce milk from the digestion of fibrous plant materials is facilitated by their symbiotic relationship with microorganisms in their rumen (Oltjen and Beckett, 1996). The rumen microbiome is a complex ecosystem consisting of bacteria, protozoa, archaea and fungi that digest material which is indigestible to their host such as plant structural carbohydrates. This is achieved through the process of anaerobic fermentation which produces volatile fatty acids (VFA), dominated by acetate (Van Soest, 1994). Acetate production is dependent on the production of hydrogen gas (H_2_) from reduced cofactors (Russell and Rychlik, 2001). In order to maintain thermodynamically favourable conditions in the rumen, this H_2_ is removed by methanogenic archaea and converted to methane (CH_4_) (Tapio et al., 2017). Methane is a potent, but short-lived greenhouse gas that makes up a significant proportion of greenhouse gas emissions from dairy production (Lorenz et al., 2019). The level of CH_4_ produced in the rumen is determined by complex relationships between VFAs, hydrogen levels and rumen microbial populations (Janssen, 2010). Generally speaking, rumen conditions that promote acetate production increase H_2_ available for methanogenesis while the production of propionate reduces H_2_ availability. Diet has the primary influence on the rumen microbiome and the resulting fermentation although host influences are also reported (Henderson et al., 2015).

In temperate grazing dairy systems, efforts are being made to reduce nitrogen (N) losses through sward species diversification (Jaramillo et al., 2021). The integration of legumes, such as white clover (*Trifolium repens*), in grazing swards has proven effective in offsetting synthetic N fertiliser use while maintaining herbage production (Murray et al., 2023). Further sward diversification through the inclusion of additional “herb” species such as plantain (*Plantago lanceolata* L.) may have the potential to further reduce N losses from grazing dairy systems through alterations to both urinary N excretion (Minnée et al., 2020) and soil N flows (Box et al., 2017; Egan et al., 2025). Differences in chemical composition between sward species, particularly in relation to fibre fractions, pectin levels and secondary metabolites have potential to influence rumen fermentation and therefore CH_4_ production (Joch et al., 2018; McCarthy et al., 2023; Waters et al., 2025). Investigations of enteric CH_4_ emissions from animals grazing more diverse multispecies (MSS) swards have, however, yielded mixed results (Jonker et al., 2019; Wilson et al., 2020; Loza et al., 2021), although, some recent research has indicated potential benefits in terms of animal performance and CH_4_ production (Jezequel et al., 2024; Woodmartin et al., 2024; Dwan et al., 2026). Woodmartin et al. (2024) noted shifts in the rumen methanogenic populations of sheep grazing herb containing swards that may be associated with reduced CH_4_ emissions. Despite this, the vast majority of previous investigation of the rumen microbiome of dairy cows has been limited to confined systems with limited investigation under grazing conditions. Furthermore, investigation of the rumen microbiome in relation to sward species diversity is even more limited, particularly in relation to methanogenesis.

Separately, animal breeding is another important aspect of grazing dairy systems which has potential to improve the environmental sustainability of milk production (Roche et al., 2018). Studies on the human gut microbiome suggest that there are significant host genetic influences on microbial populations (Benson et al., 2010; Tabrett and Horton, 2020). In ruminants, host influence on the rumen microbial population may act directly through host–microbe interactions or indirectly due to genetic differences that influence intake behaviour, rumination and digestibility. Evidence for host influence on the rumen microbiome of dairy cows is currently weak. However, studies have observed a breed effect on the microbiome of beef cattle of different pedigree, notably in their methanogenic communities (Roehe et al., 2016; Fan et al., 2021). Furthermore, the proportion of different cattle breeds that make up a particular animal’s genetics has an influence on that animals CH_4_ output (Crowley et al., 2024). Moreover, recent research in dairy cows has demonstrated that breeding for improved animal performance and feed efficiency can reduce the intensity of CH_4_ emissions relative to milk solids (MSo) produced (Yan et al., 2010; Lahart et al., 2024). A well-documented strategy to achieve improved feed efficiency in grazing systems is the crossbreeding of Holstein-Friesian (HF) dairy cows with Jerseys (Prendiville et al., 2009; Evers et al., 2023). Holstein-Frisian × Jersey crossbreds (JFX) achieve this through greater intake capacity and increases diet digestibility (Berry et al., 2018). Although such traits could potentially be related to the rumen microbiome, research has been limited in a grazing context. Therefore, in order to improve our understanding of the mechanisms driving feed and environmental efficiency in grazing dairy systems, investigation of the rumen microbiome in dairy cows of different genotypes is required.

The objectives of this study were therefore to compare the rumen bacterial and methanogenic archaeal communities of HF and JFX dairy cows grazing a grass monoculture, a binary mix of grass and white clover, or a MSS sward comprised of grasses, clovers and herbs and to relate the observed differences to methane production and intensity within such systems. We hypothesise that sward type and breed will influence the composition of the rumen microbiome of grazing dairy cows.

## 2 Materials and methods

The current experiment was undertaken in 2023 at Curtin’s Research Farm at the Teagasc, Animal and Grassland Research and Innovation Centre, Moorepark, Fermoy, Co. Cork, Ireland. All experimental procedures on the animals in this study were approved by the Health Products and Regulatory Authority of Ireland under the project authorization AE19132/P176. The study was part of a larger farm systems study described by Dwan et al. (2026) and Jezequel et al. (2024) that consisted of 48 Holstein-Friesian (HF; *n* = 24) and Holstein-Friesian × Jersey (JFX; *n* = 24) cows grazing three different sward systems; Perennial ryegrass (*Lolium perenne*) receiving 250 kg N/ha/yr. (PRG; *n* = 16), PRG and white clover receiving 125 kg N/ha/yr. (PRGWC; *n* = 16) and an 8 species mix receiving 125 kg N/ha/yr. (MSS; *n* = 16). The MSS consisted of PRG, timothy (*Phleum pratense* L.), red fescue (*Festuca rubra* L.), white clover, red clover (*Trifolium pratense* L.), alsike clover (*Trifolium hybridium* L.), plantain and chicory. Cows were assigned to each sward system at the beginning of the experiment described by Jezequel et al. (2024) and were balanced for breed, parity, calving date, milk production, milk composition, bodyweight and body condition score. All sward systems were managed within set rotational grazing systems with daily herbage allocations to achieve a target post grazing sward height of 4.0 to 4.5 cm. Daily herbage allocations were controlled with a temporary electric fence as described previously by Jezequel et al. (2024). The animal measurements have been outlined previously by Dwan et al. (2026). In brief, there were two 14-day measurement periods; from the 26th of July to the 8th of August and the 27th of September to the 10th of October. Methane emissions were measured using three Greenfeed units (C-lock Inc.) that were swapped between the treatments within each measurement period such that each treatment spent an equal length of time with each unit. The cows had a target concentrate supplementation of approximately 1 kg from the Greenfeeds per day. The composition of the concentrate was barley (250 g/kg), corn gluten (260 g/kg), beet pulp (350 g/kg), soyabean meal (110 g/kg), and minerals plus vitamins (30 g/kg). Milk production was measured daily, and milk fat and protein content were measured from subsequent AM and PM milk samples twice during each measurement period. Dry matter intake was estimated using the ytterbium oxide technique as described by Pérez-Ramírez et al. (2012). All measurements were then averaged per day across each measurement period.

### 2.1 Rumen fluid

Rumen samples were collected once from each cow at the end of each measurement using a transeosophageal rumen scoop (FLORA; Guelph, ON, Canada). All animals were offered a fresh allocation of pasture on the evening prior to rumen sampling. Samples were then collected the following morning immediately after milking at approximately 0830 h. A 4 mL aliquot of rumen fluid was then added to 1 mL of 50% trichloroacetic acid before storing at −18 °C. A further 250 mL aliquot was snap frozen in liquid nitrogen and stored at −80 °C. In the interest of animal welfare, only one attempt to collect rumen fluid was carried out on each animal at each collection. Insufficient fluid was collected from a number of cows leaving 35 animals with samples available from both periods for further analysis (PRG = 12, PRGWC = 11, MSS = 13, HF = 16, JFX = 20). The main blocking factors remained balanced after the loss of subjects and sample size was sufficient to detect differences in dietary and breed treatments based on previous literature (Noel et al., 2019; Kjeldsen et al., 2024). The acidified samples were analysed for total VFA and individual VFA (acetic, propionic, butyric, valeric, isobutyric and isovaleric acids) proportions as well as ammonia concentration as per the methods described by McAuliffe et al. (2022).

### 2.2 DNA extraction, library preparation and sequencing

The 250 μL rumen fluid samples stored at −80 °C were defrosted on ice and DNA was extracted using the repeated bead beating and column purification method as described by Yu and Morrison (2004). In brief, 1 mL of lysis buffer was added to the rumen fluid which was then homogenised for 3 min at maximum speed in a bead beater. They were then incubated at 70 °C for 15 min before being centrifuged. Supernatant was then removed to an Eppendorf tube and 300 μL of lysis buffer was added to the remaining material. The previous steps were then repeated on the remaining sample. Contaminants were precipitated from the Eppendorf tubes using 260 μL of ammonium acetate, incubation on ice followed by centrifuging. The supernatant was then removed to a fresh Eppendorf. Nucleic material was precipitated using isopropanol and the remaining supernatant was removed and discarded. DNA was then purified using Qiagen kits. Negative controls were not deemed necessary due to the high biomass nature of the samples. DNA quality was assessed on agarose gels and DNA concentration was quantified using a Nanodrop 1,000 spectrophotometer. The DNA samples were then standardised to a volume of 25 μL at a concentration of 20 ng/μl of DNA per sample. They were then shipped on ice to Novogene (UK) Co., Ltd. for PCR and sequencing.

Amplicons were generated targeting the V4 region of the 16S rRNA gene using the 515F/806R primers on an Illumina NovaSeq 6,000 sequencing system using paired sequencing (2 × 250 bp). Sequencing libraries were generated and indexes were added. The library was checked with Qubit and real-time PCR for quantification and bioanalyzer for size distribution detection. Quantified libraries were pooled and sequenced on Illumina platforms, according to effective library concentration and data amount required. Paired-end reads were merged using FLASH (Magoč et al., 2011). Quality filtering on the raw tags was performed using fastp (Bokulich et al., 2012). The SILVA database^1^ (138.1), was used as a reference to detect chimeric sequences. Chimeric sequences were removed using the vsearch package (Edgar et al., 2011). Effective tags were cleaned using DADA2 to obtain initial amplicon sequence variants (ASVs). Taxonomic annotation was performed using QIIME 2 software firstly with the SILVA database. For sequences that could not be annotated with SILVA, NCBI was used to supplement the taxonomic information. Chloroplast and mitochondrial reads were removed. Following this, ASVs were separated into their individual Kingdoms (Bacteria and Archaea). The archaeal ASVs were further classified against the RefSeq database^2^ using DADA2 in R to determine the proportion belonging to the *Methanobrevibacter* SGMT (*smithii, gottschalkii, millerae,* and *thaaurei*) and RO (*ruminantium* and *olleyae*) clades as defined by St-Pierre et al. (2015). Separate ASV tables were then constructed for the Bacteria and Archaea.

### 2.3 Data and statistical analysis

For alpha diversity analysis the bacterial and archaeal ASVs were each rarefied to their respective minimum sequencing depths (23,409 and 418 reads respectively) which were checked for suitability using rarefication curves (Supplementary Figure 1). Following this observed ASVs and Shannon diversity were calculated for each sample’s respective bacterial and archaeal populations using the phyloseq package in R (McMurdie and Holmes, 2013). Further analysis of bacterial and archaeal population composition was performed at the genus level and the clade level in the case of *Methanobrevibacter*. The non-rarefied reads were aggregated to the genus and clade levels. Principal components and the Procrustes test were used to check that aggregated data was similar in structure to the original non-rarefied data (*p* < 0.001) using the vegan R package (Oksanen et al., 2007). The aggregated bacterial and archaeal data were rarefied to their new respective minimum read depths (16,628 and 418 reads for bacteria and archaea respectively) which were checked for suitability using rarefaction curves (Supplementary Figure 1). Only bacterial genera present in greater than 5% of samples were included in beta diversity analysis in order to reduce zero inflation and increase power for detecting biologically meaningful differences.

Bacterial alpha diversity values were compared between sward systems and breeds for each measurement period separately using the Kruskal-Wallace test. Principle co-ordinate analysis (PCoA) based on Bray-Curtis dissimilarities were generated to visualise differences in bacterial beta-diversity at the genus level. The adonis2 function from the vegan package in R was used to test for statistical differences in beta-diversity between sward systems and breeds for each measurement period separately. Analysis of composition of microbiomes test was carried out on the non-rarefied bacterial ASVs present in >5% of samples. Differences were generated at the genus level between sward systems and breeds with measurement period as a fixed effect using the ANCOM-BC package in R with the Benjamini-Hockberg adjustment (Lin and Peddada, 2024).

The ratio of archaeal to bacterial reads, archaeal alpha-diversity and the relative abundance of the archaeal genera and clades were analysed using the mixed procedure in SAS with the fixed effects of sward system, breed, measurement period and their interactions (Woodmartin et al., 2024). A linear mixed model was used, as all archaeal data were normally distributed and taxonomic diversity was low both at the genus (three genera) and clade levels (two clades). Individual animal was included as a random effect with measurement period as a repeated measure and a fixed effect. A compound symmetry correlation structure was applied as it provided the best model fit as assessed by the Akaike information criterion value. The residuals of each model were checked for normality and homoscedasticity. Other effects tested were parity and calving day of the year and all potential interactions, however, none of these were included in the final model as they had no significant effect on any variable tested (*p* > 0.05). Results were expressed as least square means and the multiple comparisons were adjusted using the Tukey–Kramer method.

## 3 Results

Grazing results, herbage chemical composition, enteric emissions, animal performance and rumen VFA results have been reported previously by Dwan et al. (2026). A total of 7,638,394 reads were generated from the 16S amplicon sequencing with an average (standard deviation) of 93,151 (21,300.3) reads per sample. Following quality filtering, merging and removal of chimeric sequences there was a total of 5,878,910 reads with a mean of 71,694 (20,037.3) reads per sample which were assigned to 19,369 ASVs with a mean of 1,354 (200.0) ASVs per sample.

### 3.1 Rumen bacterial community

**Figure 1:**
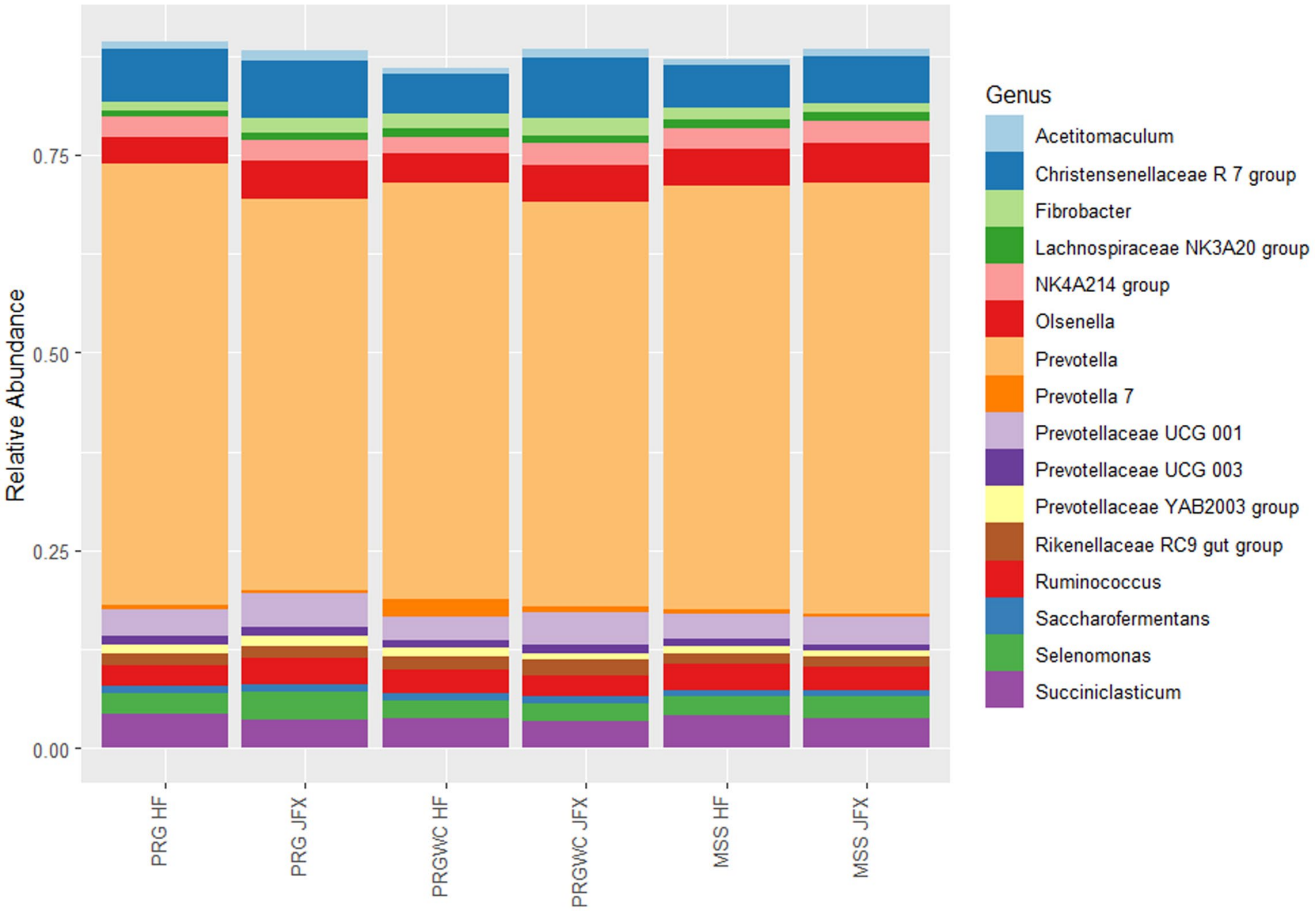
Mean relative abundance of the most abundant bacterial genera (>1%) in the rumen of Holstein-Friesian (HF) and HF X Jersey (JFX) dairy cows grazing perennial ryegrass (PRG), PRG and white clover (PRGWC) or a multispecies sward (MSS).

**Figure 2:**
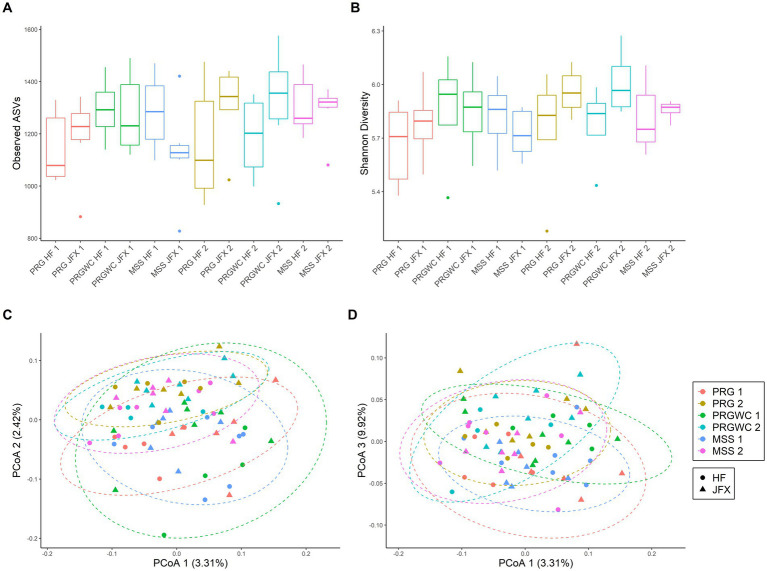
Microbiome alpha diversity analysis of rumen fluid samples from spring calving Holstein-Friesian (HF) and Holstein-Friesian × Jersey (JFX) dairy cows grazing three sward systems; perennial ryegrass (PRG), PRG and white clover (PRGWC) or a multispecies sward (MSS) in two different measurement periods (1 = summer and 2 = autumn). Observed bacterial ASVs showed no differences between sward systems and breeds. (**A**) Bacterial Shannon diversity was greater in JFX compared to HF in measurement period 2. (**B**) Principal coordinate analysis (PCoA) based on Bray-Curtis dissimilarities at the genus level plotted on the first and second (**C**) or the first and the third (**D**) principal axes showed slight separation between breeds within each measurement period.

In all samples *Prevotella* was the dominant bacterial genus (52.8%) followed by *Christensenellaceae R7 group* (6.29%), *Olsenella* (4.38%), *Succiniclasticum* (3.81%) and *Prevotellaceae UCG 001* (3.56%). The mean relative abundance of the most prevalent genera are displayed in Figure 1. Alpha diversity of the three sward systems and two breeds across measurement periods are shown in Figures 2A,B. There was no effect of sward system or breed on Observed ASVs in both measurement periods. Sward system had no effect on Shannon diversity while JFX were greater than HF in the second period only (*p* < 0.05; Figure 2B). The PCoAs are shown in Figures 2C,D. They do not show a clear separation across sward systems or breeds but show clustering by measurement period. PERMANOVA analysis revealed that the beta diversity of HF and JFX tended to differ in period 1 (*R*^2^ = 0.051; *p* = 0.009) and significantly differed in period 2 (*R*^2^ = 0.069; *p* < 0.05).

Analysis of composition of microbes identified only two genera that were differentially abundant between PRGWC and PRG, *Lachnospira* (*p* < 0.001) and the *Prevotellaceae Ga6A1 group* (*p* < 0.05) both of which were more abundant in PRGWC. Both of these genera were also more abundant in MSS compared with PRG (*p* < 0.001 and *p* < 0.01 respectively). *Pseudoramibacter* was also more abundant in MSS compared with both PRG and PRGWC (*p* < 0.01). Four genera, *Kandleria*, *Schwartzia*, *unidentified Absconditabacteriales (SR1)* and *Prevotella 7* were less abundant in JFX compared to HF (*p* < 0.01, *p* < 0.05, *p* < 0.05 and *p* < 0.05 respectively). A further three genera approached significance for lower abundance in JFX, *Prevotellaceae YAB2003 group* (*p* = 0.051), *Colidextribacter* (*p* = 0.051) and *Eubacterium cellulosolvens group* (*p* = 0.052). No genera were more abundant in JFX compared with HF.

### 3.2 Rumen archaeal community

The effect of sward system and breed on the rumen archaeal community are presented in Table 1. Neither variable had any effect on the ratio of Archaea to Bacteria. There was an interaction between sward system and breed for Observed ASVs (*F* = 3.77; *p* < 0.05) although no Tukey–Kramer adjusted pairwise comparisons were significantly different. Shannon diversity was greater (*F* = 4.91; *p* < 0.05) in MSS (1.95) compared with PRG (1.83) and PRGWC (1.81) while breed had no effect (*F* = 0.94). The relative abundance of *Methanobrevibacter* was greatest (*F* = 22.89; *p* < 0.001) in PRG (96.2%), intermediate in PRGWC (92.8%) and lowest in MSS (90.1%). There was also an interaction with breed (*F* = 3.59) where *Methanobrevibacter* was greater in JFX compared to HF in the MSS sward system only (*p* < 0.05). *Methanosphaera* was the opposite of *Methanobrevibacter*, lowest (*F* = 23.29; *p* < 0.001) in PRG (3.57%), intermediate in PRGWC (6.72%) and greatest in MSS (9.54%). *Methanosphaera* was also greater (*F* = 5.81; *p* < 0.05) in HF (7.49%) compared with JFX (5.73%). *Methanomassiliicoccus* abundance did not differ between sward systems or breeds and made up a very small proportion of total archaeal reads (0.37%). There were no differences between the relative abundances of the *Methanobrevibacter* clades, SGMT and RO, between the sward systems or breeds. On average they made up 71.6 and 18.9% of the total archaeal population across all samples, respectively.

**Table 1:** The effect of sward system (S) and dairy cow breed (B) on the least square means of the ratio of archaeal reads to bacterial reads, alpha diversity, and the relative abundance of archaeal genera and *Methanobrevibacter* clades.

	PRG		PRGWC		MSS		SE	*p*-value		
	HF	JFX	HF	JFX	HF	JFX		S	B	S × B
Archaea:Bacteria	0.028	0.028	0.020	0.026	0.028	0.022	0.0047	0.38	0.87	0.51
Alpha diversity										
Observed ASVs	9.50	9.83	8.88	10.57	11.29	9.42	0.790	0.51	0.92	<0.05
Shannon	1.84	1.83	1.80	1.83	2.01	1.88	0.055	<0.05	0.34	0.17
Genus (%)										
*Methanobrevibacter*	95.5	96.8	93.2	92.4	88.1	92.1	1.08	<0.001	0.053	<0.05
*Methanosphaera*	4.34	2.79	6.73	6.72	11.40	7.68	1.063	<0.001	<0.05	0.16
*Methanomassiliicoccus*	0.14	0.42	0.06	0.87	0.50	0.20	0.0029	0.76	0.20	0.09
Clade (%)										
SGMT	72.4	74.4	72.3	74.2	65.9	70.4	3.94	0.19	0.31	0.90
RO	20.7	18.6	19.7	16.1	19.7	18.8	3.87	0.87	0.41	0.92

PRG, perennial ryegrass; PRGWC, PRG and white clover; MSS, multispecies sward; HF, Holstein-Friesian; JFX = HF × Jersey; SGMT, *Methanobrevibacter smithii*, gottschalkii, millerae, and thaaurei; RO, *Methanobrevibacter ruminantium* and olleyae.

## 4 Discussion

The principal objectives of this study were to investigate the effects of sward species diversity and dairy cow genotype on the rumen microbial community in order to better understand their influences on CH_4_ production. The CH_4_ emissions and rumen fermentation characteristics of the HF and JFX cows grazing the three swards in this study have been described previously (Dwan et al., 2026). The main findings of that study were a reduction in CH_4_ yield from cows grazing MSS which was found to be associated with reduced rumen acetate and increased rumen butyrate concentrations. The JFX cows also had increased rumen butyrate concentrations but at the expense of propionate which resulted in no difference in CH_4_ yield compared with the HF cows. The JFX were, however, more feed efficient and produced less CH_4_ per kg of MSo. An important aspect of the current investigation is understanding the role of the rumen microbiome in these sward and animal differences.

### 4.1 Sward system

Consistent with previous research, the rumen bacterial populations in this study were dominated by *Prevotella* (Henderson et al., 2015; Betancur-Murillo et al., 2023). The other most abundant genera, *Christensenellaceae R7 group*, *Olsenella*, *Succiniclasticum* and *Prevotellaceae UCG 001*, are consistent with previous research in grazing dairy cows (Smith et al., 2020; Dwan et al., 2025). Previous research has observed minor differences in the rumen bacterial community structure (beta diversity) of PRG and PRGWC swards (Smith et al., 2020, Dwan et al., 2025) particularly in the autumn. However, sward system had no effect on bacterial beta diversity in the current study. This may be because the diets were too similar to detect a significant difference in beta diversity because although there was the addition of clover and herbs to PRGWC and MSS respectively, both swards were dominated by PRG (≥ 60% DM). It is however, surprising that greater differences in the rumen microbiomes between the cows grazing the three sward systems were not observed considering the reductions in CH_4_ yield and increased rumen butyrate concentrations associated with MSS (Dwan et al., 2026). There were, however, some minor differences in the abundance of some individual genera.

The greater abundance of *Lachnospira* and *Prevotellaceae Ga6A1 group* in both PRGWC and MSS compared to PRG suggests that these genera are associated with clover inclusion in the sward, as it was common amongst PRGWC and MSS compared with PRG. *Pseudoramibacter* may be associated with plantain or chicory as it was more abundant in MSS compared to the other two swards. There is limited information in the literature to suggest why *Prevotellaceae Ga6A1 group* or *Pseudoramibacter* would associate with the inclusion of clover or herbs in the diet of ruminants. *Lachnospira,* increased in abundance with sward diversity and has been previously associated with animals grazing white clover (Bowen et al., 2018; Smith et al., 2020). It was also associated with greater total VFA concentrations in the current study. *Lachnospira* is a pectinolytic bacterial genus found in the rumen, that produces acetate, formate, ethanol, CO_2_, methanol and small amounts of H_2_ (Cornick and Stanton, 2015). Pectin is a complex water-soluble polysaccharide found in the cell walls and intercellular tissue of dicotyledonous plants (Mudgil, 2017). There is very little pectin content in grasses although it makes up between 5 and 12% of the dry matter content of pasture legumes such white and red clover (Chesson and Monro, 1982) as well as the majority of the DM content in chicory (Sun et al., 2008). This potentially explains the greater *Lachnospira* abundance that we observed for the cows grazing PRGWC and MSS, although it is not possible to reach a conclusion based on the current analysis.

In addition to *Lachnospira*, many common rumen bacterial genera including *Prevotella, Butyrivibrio, Fibrobacter and Ruminococcuss,* possess pectin methyl-esterases that release methanol from pectin in the rumen (Kelly et al., 2019). Methanol is a substrate for methylotrophic methanogenesis which in ruminants, is largely associated with methanogens in the *Methanosphaera* genus (Tapio et al., 2017). Binary mixes of PRG with clovers, plantain or chicory have been previously associated with greater rumen *Methanosphaera* abundance compared with PRG monocultures (Smith et al., 2020; Woodmartin et al., 2024). This was also confirmed in the current study where *Methanosphaera* abundances were almost double and triple that of PRG in PRGWC and MSS, respectively. Relative to other methanogens, *Methanosphaera* has been associated with environments with reduced H_2_ levels (Poulsen et al., 2013), which may explain why the cows grazing MSS had reduced CH_4_ yield (Dwan et al., 2026). However, differences in CH_4_ yield can be better understood through the examination of gene pathways associated with hydrogen and CH_4_ production rather than taxonomic constitution (Kittelmann et al., 2014; Shi et al., 2014). Research in sheep phenotypically divergent in CH_4_ output reported no differences at microbial community level, however, there were differences in the expression of genes related to butyrate formation that were associated with reduced CH_4_ yield (Kamke et al., 2016). This is consistent with the butyrate and CH_4_ yield data related to the current experiment (Dwan et al., 2026). Future research using meta-transcriptome analysis would improve our understanding of CH_4_ yield from grazing dairy cows, particularly from animals grazing swards with different botanical compositions.

### 4.2 Breed

In grazing dairy systems, the majority of Jersey genetics are crossed with Holstein-Friesian (Berry et al., 2018; LIC Ltd. and DairyNZ Ltd, 2024). Despite this, the current study is amongst a few investigations into the rumen microbial community of JFX dairy cows. Beecher et al. (2014) and Bainbridge et al. (2016) noted no difference in the rumen microbiome of HF and JFX animals with less sophisticated analysis methodologies to the current study. Minor differences in beta diversity were observed between the rumen microbiomes of the two breed groups in the current study. Several studies comparing the rumen microbiomes of purebred Jersey and HF dairy cows in indoor total mixed ration (TMR) systems have reported a greater difference in beta diversity (Noel et al., 2019; Islam et al., 2021; Olijhoek et al., 2022; Wang et al., 2024). They also noted that on a TMR diet, Jersey cows had greater CH_4_ yield than HF cows. In grazing systems, no difference in CH_4_ yield has been reported for both Jersey and JFX cows compared to HF cows (Deighton et al., 2011; Lahart et al., 2024) which was confirmed by our recent study (Dwan et al., 2026). In that study, the main differences reported between JFX and HF dairy cows were greater feed efficiency (MSo yield per kg of DMI), greater intake capacity (DMI per kg of bodyweight) and greater proportions of butyrate as opposed to propionate in their rumen fluid (Dwan et al., 2026). There is little evidence in the literature to suggest that the minor differences in the rumen microbial community observed between HF and JFX are associated with JFX’s greater feed efficiency. However, there is some evidence that the differences in the microbiome of the two breeds potentially support the observed differences in rumen VFA concentrations (Dwan et al., 2026).

Jersey crossbred dairy cows have also been associated with improved diet digestibility, particularly for fibre (Aikman et al., 2008; Beecher et al., 2014). Fibre digestion is associated with lower propionate levels and greater rumen H_2_ concentrations (Janssen, 2010). The bacterial genera *Kandleria, Schwartzia*, and *Prevotella-7* were less abundant in JFX compared with HF in the current analysis. *Kandleria* produces lactate that is largely converted to propionate in the rumen (Ungerfeld, 2020). *Schwartzia* ferments succinate to propionate (van Gylswyk et al., 1997) while *Prevotella-7* is also predicted to be a propionate producer (Hitch et al., 2022). Reduced abundance of genera that produce propionate in the rumen is associated with increased CH_4_ yield (Danielsson et al., 2017; Dwan et al., 2025). As rumen propionate production is inversely related to H_2_ production, these results would suggest a greater supply of H_2_ in JFX relative to HF which would theoretically increase CH_4_ yield (Janssen, 2010). Despite this, JFX did not have greater CH_4_ yield (Dwan et al., 2026) suggesting that JFX cows potentially possess alternative hydrogen sinks in their rumens, however this cannot be decerned from the data in the current investigation. The physiological differences in JFX that are driving these microbial differences are not clear from the current analysis. A potential cause may be rumination time, which Prendiville et al. (2010) reported to be reduced in JFX compared with HF. Reduced rumination times are associated with lower rumen propionate concentrations and increased abundance of some *Methanobrevibacter* strains although, not with CH_4_ production (Castaneda et al., 2025). This is consistent with findings of the current study. Furthermore, animals with Jersey genetics are also reported to have reduced rumen retention times (Aikman et al., 2008, Beecher et al., 2014), and therefore increased rumen passage rates compared with HF. Greater rumen passage rates, may reduce the pool of H_2_ available for methanogenesis in the rumen (Janssen, 2010), potentially offsetting the CH_4_ promoting characteristics observed in the rumen microbiome of JFX. The relationship between the rumen microbiome, grazing behaviour and digestive kinetics of JFX dairy cows requires further investigation particularly in relation to their influences on methanogenesis and feed efficiency. More in depth meta-transcriptome analysis would be beneficial in identifying the metabolic pathways associated with factors such as methanogenesis, hydrogen metabolism and butyrate synthesis.

## 5 Conclusion

The results from this study support previous findings that the core rumen microbiome of dairy cows is largely robust to differences in sward composition. However, the results also show that increasing sward diversity through the inclusion of clovers, plantain and/or chicory increases the abundance of pectinolytic bacterial genera and the methanogenic archaea genus, *Methanosphaera*. These results also indicate that HF and JFX dairy cows have slightly different rumen microbiomes with a greater abundance of bacterial genera associated with propionate production and the archaeal genus *Methanosphaera* in HF compared with JFX. Although this research identified both sward and breed influences on the rumen methanogenic community, it is not possible using data generated form 16S amplicon sequencing to directly demonstrate whether this functionally influenced methanogenesis. More in-depth investigation into microbial gene expression are required to better understand these relationships.

## Data Availability

The datasets presented in this study are publicly available. This data can be found at: https://www.ncbi.nlm.nih.gov/sra/ PRJNA1465808, accession number PRJNA1465808.

## Footnotes

1
https://www.arb-silva.de/

2
https://zenodo.org/record/2541239
