# Supplementary material for: Understanding the influence of sward type and dairy cow breed on enteric methane emissions through investigation of the rumen microbiome
Source: Front Microbiol. 2026 Jun 17;17:1799911. doi: 10.3389/fmicb.2026.1799911 (PMC13322137; doi:10.3389/fmicb.2026.1799911)
Supplement: Supplementary file 1 [file Image_1.pdf]

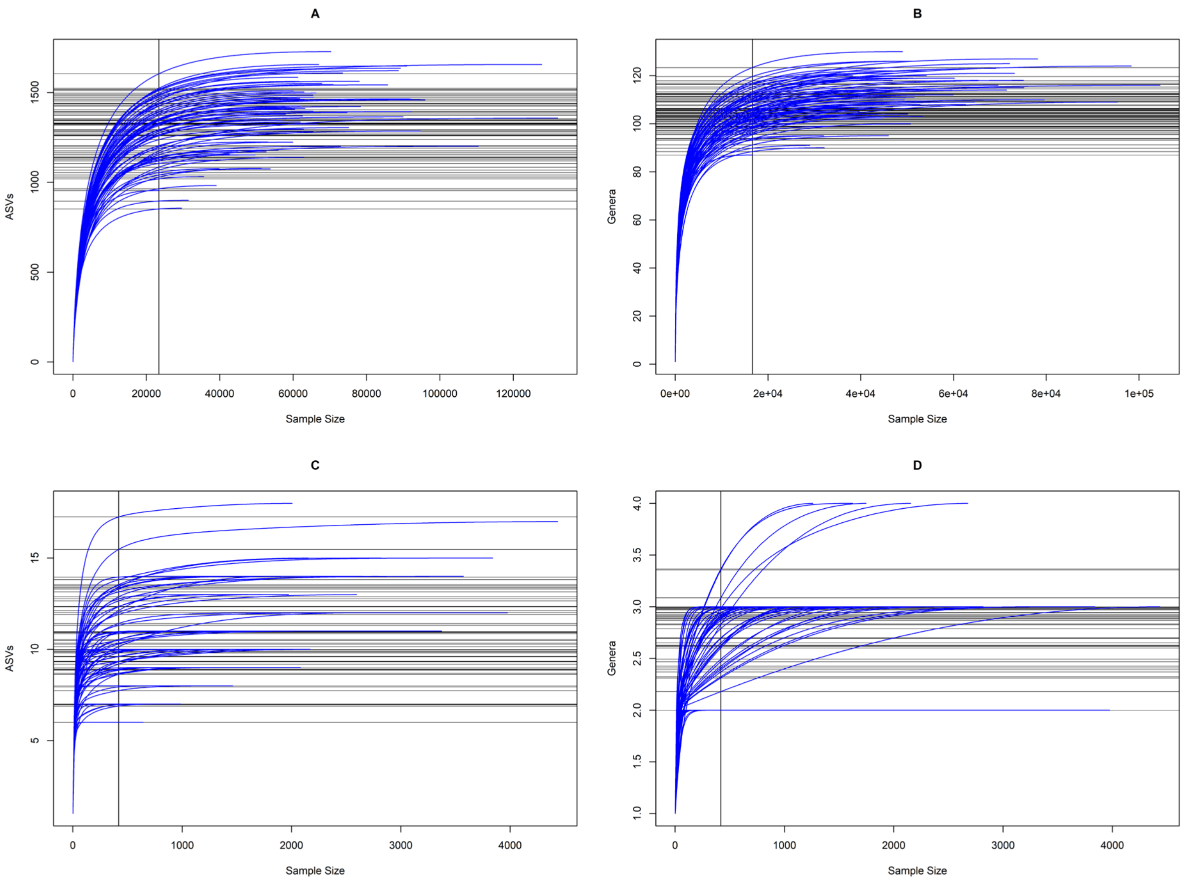

Supplementary Figure 1.

Rarefication curves for (A) bacterial ASVs, (B) bacterial genera, (C) archaeal ASVs and (D) archaeal genera.
